# Azoturia1Read at the Annual Meeting of the Pennsylvania State Veterinary Medical Association, Philadelphia, March, 1899.

**Published:** 1899-05

**Authors:** Otto G. Noack

**Affiliations:** Reading, Pa.


### AZOTURIA.[1]

By Otto G. Noack, V.S.,

READING, PA.

Azoturia is a disease described under as many names as there are opinions of its cause and origin. Even our best authorities

[1] Read at the Annual Meeting of the Pennsylvania State Veterinary Medical Association, Philadelphia, March, 1899.

differ, each of them having a different theory and separate name for it.

The French authors call the disease hæmoglobinuric paraplegia, and, like Cadiot, say that it is an infectious disease caused by intestinal microbes, which, under the influence of physico-chemical modifications of the medium, acquire an abnormal virulence and secrete poisons of extreme activity. Lasartesse claims that azoturia followed sometimes lymphangitis, and Ligneres that he discovered the bacillus. Some call it a liver and kidney disease. In Friedberger and Fröhner (Zuill's translation) hæmoglobinæmia and hæmoglobinuria are explained in this way. When the striated muscles are exposed to violent irritations, phenomena of decomposition are produced in their substance, in the course of which the coloring-matter of the muscle, which is identical with hæmoglobin, becomes free and passes into the blood. Among these irritations external cold plays a most important part; it increases in a reflex manner the organic transformations. The very great majority of cases of hæmoglobinuria are traced to no other cause. On the other hand, Bollinger denies cold to be the cause of this disease, and says that all changes of the blood, skeleton-muscles, and liver in hæmoglobinuria show that a poison or an agent resembling poison is the cause of this pathological process, showing its effects first on the blood, which produces a fatal hæmoglobinuria and nephritis. This toxic agent is taken into the system with the food. Later, however, this author modified his theory by stating that the poison is produced by the effects of work and cold, causing thus an auto-intoxication. Dieckerhoff defines azoturia as an acute disease of the whole system, which is characterized principally by a certain parenchymatous inflammation of the skeleton-muscles and by bloody infiltration and painful irritation of the marrow, and complicated with hæmoglobinuria and acute nephritis. Its cause is given as auto-intoxication produced by fermentation of too much protein. In the *Thieraerztliche Wochenschrift*, 1896, he came to the conclusion that the overproduction of lactic acid caused the intoxication. This opinion was proven prior to his statement, and affirmed by experiments and examinations of Lehman, late professor at Leipsic. Azoturia, therefore, must be defined as a disease caused by auto-intoxication by lactic acid, arising from disturbances in nutrition caused by amylaceous food and by rest peculiar to the horse.

To understand, however, how such auto-intoxication in a being can occur and what facilities there are for prevention, we must consider the animal's system as a very fine laboratory where chemical processes are taking place continuously which are kept up by the introduction of nutriment, a compound of chemicals. The system, therefore, is more or less exposed to auto-poisoning, as the nutriment may contain something for the production of substances injurious to one or the other part of this laboratory.

There are four ways to eliminate the overproduced, obnoxious material from the system. First, the stools, but by the slow movement of the intestines the mucous membranes is given ample time to absorb such a quantity of the poisons as to injure vital parts. The cutaneous emunction of detrimental matters is of great importance, as poisonous gases and salts leave the system in this manner. The nitrogen is mostly ejected by the pulmonary outlet, the organ for oxydizing the blood. The last and most important way of excreting the poisons out of the system is by the kidneys in connection with the liver. The function of these organs is to cleanse the blood and prevent destruction of the animal life, if possible. Consequently these organs become sufferers in such cases through overwork, and have to undergo pathological changes. If the surplus of a poison is so great that these organs are not able to free the blood from it, all the vital parts will become affected, and death will be the result.

We have seen thus far how poisons may be produced and how the system is protected. Now we have to become acquainted especially with the production, presence, and chemico-physiological properties of lactic acid in the system. Lehman, in his manual of *Chemical Physiologie*, gives the following description: Lactic acid is very frequently but by no means always contained in the gastric juice, together with hydrochloric acid; in the saliva it is only recognized with certainty in diabetes. The acid reaction which the contents of the duodenum and jejunum exhibit, especially after vegetable diet, depends in a great measure on lactic acid; lactate of lime is often found in the duodenum of herbivorous animals. The contents of the large intestine also, which exhibit so strong an acid reaction after amylaceous and saccharine food, owe this to the lactic acid developed by fermentation, and accompanied by butyric acid. This acid may be detected in the chyle of the thoracic duct of the horse, especially after feeding on oats and starch; it is probable, according to some observations, that it also exists in the lymph.

Lactic acid is not to be detected in the blood in normal conditions, as it is there quickly oxidized and entirely consumed, whether introduced from the intestines or other organs; lactates of the alkalies injected into it disappear rapidly and show themselves in the urine as carbonates. In pathological blood, however, especially in puerperal fever and leukæmia, it is detected beyond doubt. Occasionally it exists in puriform exudations which have remained long in abscess-cavities. This acid is not contained in healthy milk; it is soon formed in it from lactin by fermentation. Lactic acid is further found in the juice both of striated and smooth muscles in tolerably large quantities. In the acid juice of the spleen also lactic acid and lactates of the alkalies are found. In the urine it is occasionally, but not constantly found. It only passes into this when it is not altogether consumed in the blood, hence we find it in the urine of animals after feeding them on amylaceous food and confining them constantly in stalls, also in disturbances of respiration by emphysema of the lungs or tuberculous deposits. Very often it is formed in the urine during fermentation.

It is obvious, from the circumstances in which lactic acid is found, that it has a double origin in the animal economy. It arises in the primæ viæ by the fermentation of the amylacea; but as it exists in the muscles in proportion to the previous energy of their action, it may be regarded as a product of the metamorphosis of the muscular fibre.

The physiological importance of lactic acid must not be too highly regarded; for in the first place it is the cause actually, with hydrochloric acid, of the digestive power of the gastric juice; no other mineral or organic acids can take the place of these two in the gastric juice. Secondly, lactic acid assists, as free acid, in the absorption or transudation of the digestive food from the primæ viæ into the alkaline blood or lymph. Thirdly, by the ready combustibility of its salts in the blood it becomes an important assistant in maintaining the animal temperature; and, finally, it excites an elastic tension in the muscles, as opposed to the alkaline blood, which possibly is not without influence on the muscular function.

This shows that plenty of lactic acid is produced in the horse's intestinal canal through the food he gets. Besides, if confinement be added to rich food the movements of the bowels is slower than if in action and the mucous membranes have time to absorb the overproduced acid which the blood cannot consume and conform in the innocuous carbonates. The free acid, therefore, has to circulate in the blood, destroying its alkalinity, as proven by Dieckerhoff. Repeated tests of samples of blood drawn from the jugular veins of azoturia cases have shown that the normal alkalinity is greatly reduced, the reaction even neutral. Consequently the tension between the blood and muscles has to disappear and the muscular function to be disturbed. The lactic acid contained in the muscles now will cause irritation and lead to degenerative conditions in the muscular fibres. If the horses are taken out at this time they will go all right for some distance, but the disorder in the muscles soon will manifest itself, the horse at first giving way in his hindparts, then knuckling over, showing great distress, and at last going down, often not being able to rise, and apparently afflicted with such intense pain that the case might be mistaken for colic; but close observation and judgment will soon note the difference. At the same time in many cases the heart is somewhat afflicted, the shock being arhythmic, pulsation and respiration accelerated, associated with profuse perspiration. In light cases the symptoms may not be so significant, but the post-mortem of all these cases reveals an affection of the heart, the right part being in a flabby, decomposed condition, which appears always when poisoned with lactic acid, as shown by Foster's experiments on dogs.

Furthermore, we find hæmoglobinuria to be in existence, which always accompanies the poisoning with acids or septic processes (Laugerhaus). As the hæmoglobin is eliminated partly through the liver, partly through the kidneys (hæmoglobinuria), we see that the urine is of an abnormal color, from dark yellow to coffee-brown, containing more or less albuminous substances and having often a syrup-like consistency; while the hæmoglobin has some trouble to pass off the small uriniferous tubules, it accumulates here if the attack is grave, and causes the urine to diminish and even anuria, at the same time giving rise to a deep nephritis, generally ending fatally. Peristalsis also is slow, and the evacuation of feces occurs sometimes with signs of great pain. In most cases with fatal result we find by autopsy a deep, parenchymatous nephritis, and the kidneys in a soft condition and in a state of decomposition. The blood looks dark, tar-like, and is not coagulated. The liver always is enlarged and swollen. Sometimes one or the other hind leg, in most cases the left, shows paretic symptoms, caused by the bloody infiltration of the spinal cord.

After we are acquainted with the symptoms and pathological changes of this disease we should consider which will be the best medical treatment. While the cause, as shown above, is a poison generating in the intestinal canal, our treatment, therefore, must be directed, first, to eliminate this poison through the ways mentined ; secondly, to neutralize it, if possible.

Nothing better can be given than aloes, which is one of the best laxatives for the horse, and at the same time a cholagogue which increases the action of the liver-cells and the secretion of this organ. If this be followed by bicarbonate of soda, which will neutralize the acid and also acts as a diuretic, the result will be more or less satisfactory. If paretic conditions are existing in the hind legs, the hot iron may be applied in the lumbar region with good effect, and if necessary it may be repeated till complete use of such member is restored.

Of fifteeen cases which I treated last year in this manner I only lost one, which showed grave pathological changes of the kidneys.

